# Comparison of Toxicological Bioassays for Whiteflies

**DOI:** 10.3390/insects11110789

**Published:** 2020-11-12

**Authors:** Tanner C. Sparks, David G. Riley, Alvin M. Simmons, Liangzhen Guo

**Affiliations:** 1 Department of Entomology, University of Georgia Tifton Campus, Bldg 4603, 110 Research Way, Tifton, GA 31794, USA; tcsparks@uga.edu; 2 USDA, Agricultural Research Service, U.S. Vegetable Laboratory, 2700 Savannah Highway, Charleston, SC 29414, USA; alvin.simmons@usda.gov; 3 Department of Biotechnology, College of Agricultural Sciences, Guangdong Ocean University, No.1 Haida Road, Zhanjiang 524088, China; guolz@gdou.edu.cn

**Keywords:** *Bemisia tabaci*, bioassay, insecticide resistance management, IRM, toxicology

## Abstract

**Simple Summary:**

Insecticides are commonly used to manage whiteflies in many crops including vegetables, but frequent use can cause these pests to become resistant to insecticides. Resistance can lead to control failure and severe crop damage, thus the need for insecticide efficacy testing and insecticide resistance monitoring. A study was conducted to determine whether any current methods of toxicity assays are better than others for testing whiteflies for insecticide resistance and efficacy for better information to make effective pest control decisions.

**Abstract:**

Two *Bemisia tabaci* populations from Georgia and Florida, USA, were tested for their response to insecticides across different toxicological bioassay methods. Five insecticides in four Insecticide Resistance Action Committee (IRAC) groups (imidacloprid (4A), dinotefuran (4A), flupyradifurone (4D), pyriproxyfen (7C) and cyantraniliprole (28)), were evaluated against a water check. The routes of application to the plant used were either leaf drench or (systemic) root drench. The four different whitefly bioassay methodologies tested were two published IRAC methods, a clip cage method, and a new tube method. A split–split experimental design was used to assess any interactions between application route, bioassay method and insecticide treatment. Application route had no significant effect on efficacy. However, bioassay method affected overall whitefly mortality, with the dish method having reduced mortality compared to other methods, except for the clip cage method. High rates of cyantraniliprole, dinotefuran and flupyradifurone insecticides resulted in the highest incidence of adult whitefly mortality. Significant interactions relative to percent adult mortality were found between the insecticide and bioassay method for both populations assayed. The clip cage method was more sensitive in terms of dose mortality response followed by the cup and tube methods. The dish method was the least responsive to insecticide dose. Other interactions are discussed.

## 1. Introduction

*Bemisia tabaci* (Gennadius) is a major pest of concern for vegetable growers in the southeastern United States. This diminutive hemipteran feeds on tomatoes, cucurbits, cole crops, beans, peppers, and numerous other crops including cotton and ornamentals [1]. Whiteflies transmit at least 300 known plant diseases [2]. Through the use of toxicological bioassays, the highly prevalent B biotype (Middle East-Asia Minor 1; MEAM1) was reported as expressing resistance to carbamates, organophosphates, pyrethroids, and imidacloprid from 1990 to 2013 [3,4,5,6,7,8]. Resistance to growth regulators such as pyriproxyfen was documented within the southwestern USA [9] and by 2011, baseline susceptibilities to the new anthranilic diamides were also being established [10]. By 2007, there was bioassay evidence that more insecticide-resistant populations were displacing the less-resistant whitefly populations in China [11]. In Florida, imidacloprid was being over used to control whitefly-transmitted plant virus, so effective insecticide resistance management programs had to be developed [12]. Clip cage bioassay data formed the basis of this program which continued on to add diamides, such as chlorantraniliprole and cyantraniliprole, to the long-standing neonic bioassay surveys [13,14,15,16,17]. Increased resistance to cyantraniliprole in the Q biotype (Mediterranean; MED) was reported in 2018 through extensive toxicological tests [18], so the use of bioassays for whitefly insecticide resistance management continues to be of worldwide importance [19].

There is very limited information of the status of whitefly insecticide resistance in Georgia, USA [20]. The majority of insecticide resistance management (IRM) information for the southeast has been generated in Florida [12,13,14,15,16,17]. In the fall of 2017 in Georgia, there were record breaking whitefly populations and extensive vegetable crop damage following a population increase in cotton [21]. This event renewed interest in regional management of whiteflies and highlighted the need for insecticide efficacy testing and insecticide resistance monitoring for this pest. The first step for this new state monitoring program was to determine which whitefly bioassay method would provide the cleanest insecticide response data for a large-scale resistance monitoring program in Georgia.

The bioassay methods previously used for monitoring resistance include treated vials for contact activity against adult whiteflies [22], clip cage on plants or cuttings that were either directly drenched [13] or had insecticide mixtures applied systemically through the leaf petiole [13,14,15,16], and similar bioassay application methods, but evaluated in a petri dish [11,17,23] or a cup [24] container. This diversity in methodology reflects in part the various physical pathways of the toxicant to the whitefly, i.e., whether the insecticide exhibits cuticular contact toxicity, toxicity through ingestion, or a combination of both. Because whiteflies are phloem feeders [25] and most insecticides used against whiteflies are currently systemic [19], most methods allowed the insecticide to be imbibed by the plant before bio-assaying. There is a need for comparison studies to evaluate the consistency between bioassay methods which allow for whitefly feeding needed further investigation relative to representative insecticides used against whiteflies.

Insecticides can be categorized as mainly systemic or contact relative to whitefly control. Examples of these include systemics such as cyantraniliprole and dinotefuran and mostly contacts such as the growth regulator buprofezin or the pyrethroid insecticide bifenthrin. Further, the action of insecticides can be quick knockdown, e.g., imidacloprid, or slow acting, e.g., insect growth regulators such as pyriproxyfen [26]. One bioassay method to compare diverse insecticide response based on a single collection of adults is a difficult goal. It was hypothesized that assessing mortality of adults along with effects on oviposition and egg hatch could provide such a multipurpose method. Thus, the objective of this study was to compare different types of methodologies for assessing adult mortality, oviposition, and nymph emergence. The null hypotheses tested were that (1) application route (drench vs. foliar application), (2) whitefly bioassay method and (3) insecticide treatment would not result in significantly differing whitefly mortality, and there would not be significant interactions between each of these factors.

## 2. Materials and Methods

Laboratory whitefly colonies of *B. tabaci* used in this study were from a field collection on cotton in Georgia during a summer outbreak in 2017 at the Coastal Plain Experiment Station at Tifton, Georgia and from a laboratory whitefly population at the Gulf Coast Research and Education Center at Wimauma, Florida in the summer of 2018. Both colonies were maintained on cotton plants in individual rearing rooms until assays for this study were initiated during the spring of 2019. Adult *B. tabaci* were aspirated into pipettes from leaves of colony plants before being introduced to bioassay arenas. All plant material used in the bioassays were either whole plant or excised leaves from untreated cotton ST4946GLB2 seed grown at 30 °C, 70% relative humidity, and 16:8 day: night cycle in growth chambers (Percival model E-36L2, Perry, IA, USA). The cotton plants in these tests were grown to the two-expanded-true-leaf stage before using. Initial whitefly mortality counts were taken one hour after introduction of adults to the bioassay arenas. This was performed to assess whitefly mortality due to handling. This initial mortality was subtracted from later mortality counts because they did not reflect the effects of treatment on the insects. Adult whiteflies were counted as dead if they met the following criteria: lack of discernible movement, obvious desiccation, and/or resting on a surface with contact made by any part of the body other than the tarsi. Whiteflies that moved or were in contact with the plant tissue in what could be described in typical feeding posture were marked as live. Counts of living and dead whiteflies were taken every 24 h and testing was concluded after 72 h elapsed. Cotton leaves used for each test were placed in labeled Ziplock bags and frozen for later egg and nymph counts. Counts were made on the whole underside of leaves with a dissecting microscope at 20× magnification.

The experimental unit in this study was the bioassay arena. Treatments were arranged in a split–split plot design with the main treatments as application route, i.e., leaf drench or systemic root treatment. Sub-treatments were the four assay methodologies used, and sub-sub-treatments were the insecticide treatments with two rates, the active ingredient at a high labeled rate and 1/10 that concentration. Specific methodologies were described in the following sections. The split–split experiment was employed to detect interactions between treatment levels.

### 2.1. Application Route (Main Plot)

Leaf dip treatments were performed by dipping cotton leaves into sub-sub-treatments for 30 s. Afterward, they were air dried under ventilation for one hour to reduce the possibility of drowning introduced whiteflies on surface water droplets. Systemic treatments were administered by filling the 20 mL scintillation vials used for the clip cage and tube methods the sub-sub-treatments. The roots of cotton plants used in these tests were trimmed to ~5 cm lengths before placing in the vials with continual access to the insecticide for 24 h before introducing whiteflies. The reservoir space in the IRAC 008 method [24] was filled with insecticide solution or water and the leaf petiole was placed inside, allowing for continued uptake into the leaf inside of the top cup. Only the IRAC 015 method [23] with the petri dish did not provide a way for continual uptake into the leaf. Excised leaves instead had their petioles placed inside of the designated sub-sub-treatment and allowed to uptake the solution for 24 h before the introduction of whiteflies to the arena.

### 2.2. Bioassay Method (Subplot)

**Figure 1:**
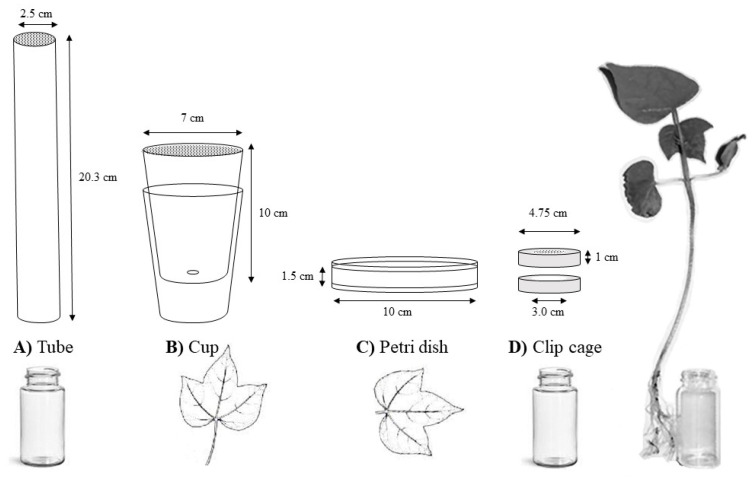
Bioassay methods for testing whitefly adult mortality: (**A**) tube and (**D**) clip cage methods used a cotton seedling; (**B**) cup and (**C**) petri dish methods used an excised cotton leaf.

Clip cages were constructed of foam pipe insulation meant for a 25.4 mm pipe by FrostKing (Thermwell, Mahwah, NJ, USA) sliced into rings with mesh screening glued on top to create a bioassay arena that could be placed over treated leaves and allow viewing of the whiteflies within (Figure 1). Aspirated adults were cooled for 5 min at 2 °C to prevent escape during introduction to the arena. Whiteflies were tapped out of the pipettes and into the clip cage before securing the clip cage to the underside of the cotton leaf using another foam ring and paperclips. Plants used in this methodology had at least one fully mature leaf and was placed in a 20 mL scintillation vial with roots washed of soil, and the vial was filled with tap water. Plants used in a leaf drench treatment had the leaf designated for whitefly feeding dipped in treatment solution, while those used in root drench had their 20 mL vial filled with 15 mL of treatment solution.

The IRAC 008 method or the “cup” method used two clear, plastic cups as the arena [23]. The two cups (Fisher Science Education™ Plastic Cups, Waltham, MA, USA) were stacked inside one another forming a space between the bottom of the two that was filled with either water or the insecticide solution that was being tested (Figure 1). A hole ~2 mm in diameter was made on the bottom of the top cup so that the petiole of an excised leaf could fit through and attain water from the reservoir without allowing the whiteflies in the top cup to fall in. Adults were tapped into the arena from the pipette and screening was placed over the mouth of the cup to prevent escape.

The IRAC 015 method required an agar gel to be made and pressing the treated leaf into the gel so that the underside was exposed out of the gel on which the whiteflies to feed [23]. Agar solution was poured into a 100 mm by 15 mm disposable polystyrene petri dish (VWR™, Radnor, PA, USA) with multiple ~0.5 mm diameter ventilation holes punched into the top lid for air flow (Figure 1). Care was taken to time the gel so that it was not so hot as to damage the leaf, but also not completely solidified to the point the leaf could not be pressed into it, i.e., still in liquid state. If not pressed into the gel enough, the leaf would fail to stay hydrated during the experiment and wither to the point that the adults would not feed on it. Leaves used for the systemic testing had their petioles placed in the treatment solution and could imbibe for 24 h before the beginning of testing. This was the only incidence in which systemic treatments were given before testing began and was performed because the methodology did not provide for continuous systemic treatment.

The University of Georgia tube method was created for testing insecticide response over an entire whitefly life cycle, i.e., adults, eggs, nymphal stages, and adult emergence. This method involved placing cotton seedling into 20 mL scintillation vials with water or insecticide as described earlier in the clip cage explanation. Due to size constraints, the plant selected with this method was the same age, but with only one true leaf fully developed. All cotyledons or budding second leaf were removed from the plant. Cotton balls were fitted around the stem in the mouth of the vial and Parafilm™ (Fisher Scientific, Waltham, MA, USA) was stretched around the cotton ball plug to lock the stem of the plant in place as well as create a waterproof seal from the vial and the plant above. A clear plastic tube 2.86 cm in diameter and 20.3 cm in length produced by ClearTec Packaging (Park Hill, MO, USA) was placed over the plant using a paper sleeve or a laboratory spatula to position the curled cotton leaf inside the tube (Figure 1). Ventilation with nylon chiffon screening was made in the side of the tube to reduce humidity and the possibility of condensation forming which trapped whiteflies inside the tube arena. Adults would stick to the plastic wall if they were wet or drowned in condensation droplets.

### 2.3. Insecticide Treatment (Sub-Subplot)

Two sets of water-treated control treatments were prepared along with 10 insecticide treatments. Five insecticide products commonly used for whitefly control were chosen: pyriproxyfen (Knack 0.86EC, IRAC Group 7C, Valent Corporation, Walnut Creek, CA, USA), dinotefuran (Venom 70SG, IRAC Group 4A, Valent Corporation, Walnut Creek, CA, USA), cyantraniliprole (Exirel 0.83SC, IRAC Group 28, FMC, Philadelphia, PA, USA), flupyradifurone (Sivanto 200SL Bayer Crop Science, Philadelphia, PA, USA), and imidacloprid (Admire Pro 4.6F (Bayer Crop Science, Research Triangle Park, NC, USA). Two concentrations of each product were used during testing: the maximum label rate for vegetables according to the Georgia Pest Management Handbook [27] and one-tenth that rate. These were pyriproxyfen 82.4 mg a.i. and 8.24 mg a.i., imidacloprid 99.2 mg a.i. and 9.92 mg a.i., dinotefuran 210 mg a.i. and 21 mg a.i., cyantraniliprole 105.4 mg a.i. and 10.54 mg a.i., and flupyradifurone 188 mg a.i. and 18.8 mg a.i. per liter, respectively. Two untreated checks were used in these experiments to measure variation within a treatment.

### 2.4. Data Analysis

Each split–split plot experiment had four replicates. The experiment was repeated twice using the whitefly populations, the Georgia and Florida laboratory colonies. All corrected percent mortality data were run through SAS Enterprise [28] using the Proc GLIMMIX procedure considering reading hour as a repeated measure. Interactions between treatment levels were measured as well as splicing each treatment into their 24 h observation points. Doing so allowed us to analyze how mortality changed over time. The total oviposition and nymph data were subjected to individual analysis of variance by using Proc GLM in SAS [28] using a spilt-split plot design with insect counts fitted to a negative binomial distribution. Significant interactions were reported and means graphed with standard errors for describing interaction effects. Main, sub- and sub-subplot means separation was tested with Tukey’s test (for repeated measures data) and LSD (for non-repeated measures data such as egg counts) (*p* < 0.05) following a significant split–split plot-level effect (*p* < 0.05).

## 3. Results

The different whitefly populations used in these tests provided experimental replication, but without a major difference between these populations in terms of overall insecticide susceptibility. The overall average % mortalities for the Florida and Georgia populations by 24, 48 and 72 h reading were 26 ± 1.3, 49 ± 1.7, 58 ± 1.8, and 36 ± 1.6, 57 ± 1.9, 68 ± 1.8, respectively. Thus, the Georgia population tended to be more susceptible by ~10%. Significant treatment effects on percent mortality tended to be the same across both populations with the only exception of the bioassay by insecticide interaction (Table 1).

### 3.1. Application Route (Main Plot)

Application route, leaf vs. root drench, did not significantly affect percent adult mortality regardless of the bioassay method or the insecticide tested for either whitefly population (Table 1). Application route did not affect oviposition counts in the Florida or Georgia population (Table 2). Differences in nymph hatch were not observed between the two application routes (Table 3). This confirmed that either application route was equally effective for assessing adult mortality using the diverse active ingredients in this study. This suggested that data generated from air-dried leaf dips are comparable to 24 h systemic applications. Since these insecticides will usually enter the whitefly through ingestion of vascular fluid, it is likely that the insecticides used in this study could penetrate plant leaf tissues. There were a few significant interactions with one or the other whitefly population, but not both, described in the section on interactions.

### 3.2. Bioassay Method (Subplot)

**Figure 2:**
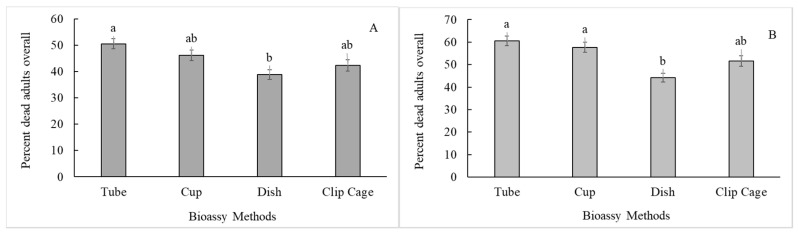
Percent adult mortality of the Florida (**A**) and Georgia (**B**) whitefly populations for tube, cup, petri dish, and clip cage bioassay methods. Bioassay means followed by the same lower case letter are not significantly different, Tukey’s test *p* < 0.05.

**Figure 3:**
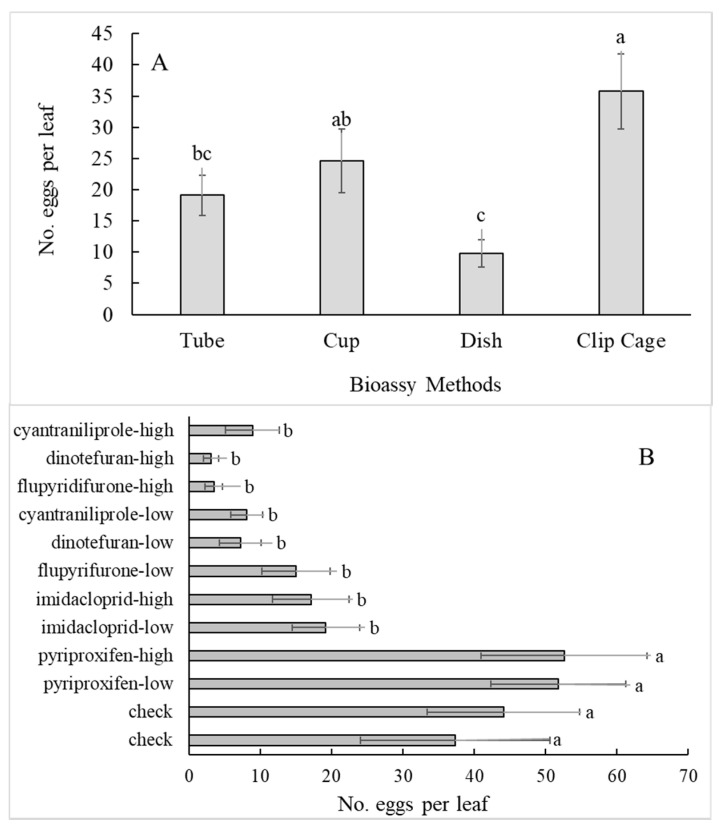
Total number of eggs observed on bioassay leaves over both whitefly populations combined across the tube, cup, petri dish, and clip cage methods (**A**) and insecticides (**B**). Means within treatment with the same lower case letter are not significantly different, LSD *p* < 0.05.

The bioassay method significantly affected adult mortality for both populations (Table 1), but not oviposition (Table 2) and nymph hatch (Table 3). Among the four methodology types, the dish method exhibited reduced mortality compared to the tube method for both populations (Figure 2A, B). Interestingly, the dish method also had the fewest eggs laid on the test leaf tissue (Figure 3A). There were several significant interactions between bioassay method and other treatment levels discussed in the section on interactions.

### 3.3. Insecticide Treatment (Sub-Subplot)

**Figure 4:**
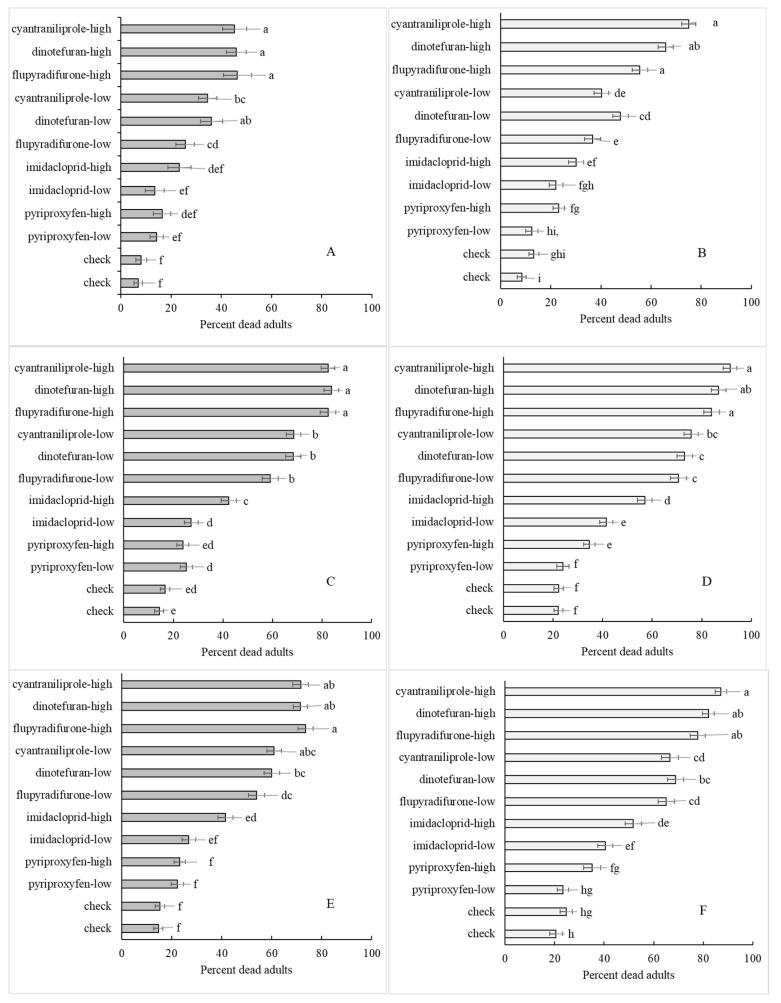
Percent adult mortality of the Florida (**A**,**C**,**E**) and Georgia (**B**,**D**,**F**) whitefly populations across insecticide treatments at 24 h (**A**,**B**), 48 h (**C**,**D**) and overall (**E**,**F**) (means with the same lower case letter not significantly different, LSD 24 and 48 h, Tukey’s test overall, *p* < 0.05).

There were significant insecticide treatments effects for both populations in terms of percent dead (Table 1) and number of eggs laid (Table 2). There was a lack of significant insecticide effects on nymph hatch (Table 3). This was likely due to the lack of sensitivity of these methods to detect nymph hatch with the short turnaround time from oviposition to nymph assessment. Adequate assessment of treatment effects whitefly nymph mortality was not indicated with this methodology and will likely need pre-infested leaves as a starting point. However, assessment of adult mortality was clearly achievable with all the methods tested at 24 and 48 h (Figure 4A–D) with a couple of caveats. There was a significant amount of initial mortality in the checks that has to be accounted, averaging approximately 15% overall, even with eliminating initial mortality as a consequence of loading the arenas (assumed to be mechanical injury unrelated to the bioassay treatments). Second, the difference in mortality due to rates was not significant for three of the five insecticides tested (Figure 4A).

Sensitivity of a bioassay methodology to rates is a desirable attribute if monitoring dose mortality response is the goal. The most efficacious insecticide treatments were the high rates of flupyradifurone, cyantraniliprole and dinotefuran, each of which had significantly higher mortality than the high rates of imidacloprid and pyriproxyfen as well as the water check, overall (Figure 4E,F). Flupyradifurone had a significant rate response overall for both the Florida and Georgia populations (Figure 4E,F, respectively). Cyantraniliprole had a significant rate response in at least one population (Figure 4E,F). Significant interactions between bioassay method and insecticide treatment discussed in the section on interactions revealed sensitivity to rates.

Neither concentrations of pyriproxyfen had significant differences in mortality or egg counts from both controls, though it did display a slightly raised mortality in adults. All other insecticide treatments showed reduced oviposition (Figure 3B). Pyriproxyfen, being an insect growth regulator (IGR), was not expected to show any mortality in adults and only affect nymph development. As previously mentioned, this methodology did not serve to assess this activity. To properly test the effectiveness of an IGR would require maintaining nymphs and observing mortality or growth rates over more time than the three days allotted for these bioassays.

### 3.4. Interactions

**Figure 5:**
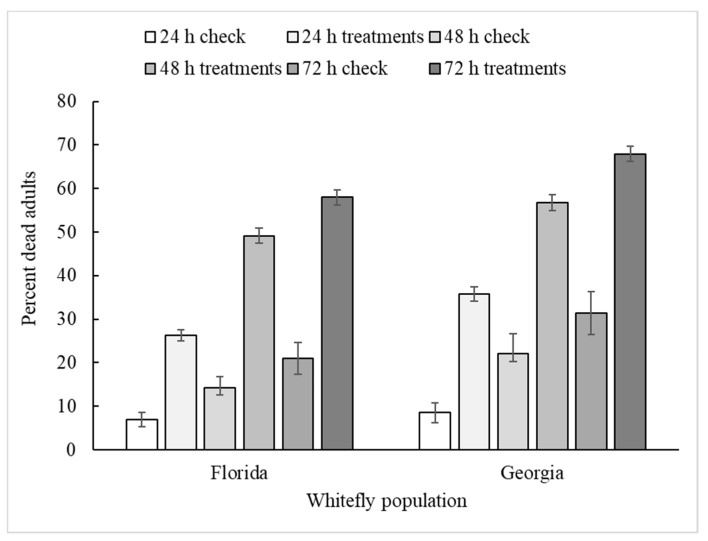
Mean % whitefly adult mortality (±se) for the untreated check and the average over all insecticide treatments at 24, 48 and 72 h for the two test populations.

The effect of time on mortality was significant and contributed to several significant interactions (Table 1). A longer insecticide exposure interval increased the likelihood for lethal effects in the adult (Figure 5). In the Georgia population, there were significant interactions between hour and three factors, application route, bioassay method and insecticide treatment (Table 1). In the Florida population, only the hour x insecticide interaction was significant (Table 1). There was an hour x insecticide interaction, which, based on the individual mean separation data, was that the differences in whitefly mortality between rates diminished for the most effective insecticides over time (Figure 4A–D). Further, the check mortality tripled from 24 h to 72 h, raising mortality rates in the less effective insecticides along with it. The average percent mortalities over both populations for the checks in tube, cup, dish and clip cage methods at 24 and 48 h were 10.2 ± 1.6, 4.4 ± 1.9, 8.6 ± 3.6, 6.0 ± 2.7 and 15.0 ± 2.2, 12.7 ± 2.6, 18.2 ± 6.2, 21.8 ± 7.0, respectively. This explains in part how the mortality percentages increased with low rates of insecticide over time (Figure 4A–D). This interaction suggests that shorter bioassay intervals might better detect insecticide rate responses across all methods.

**Figure 6:**
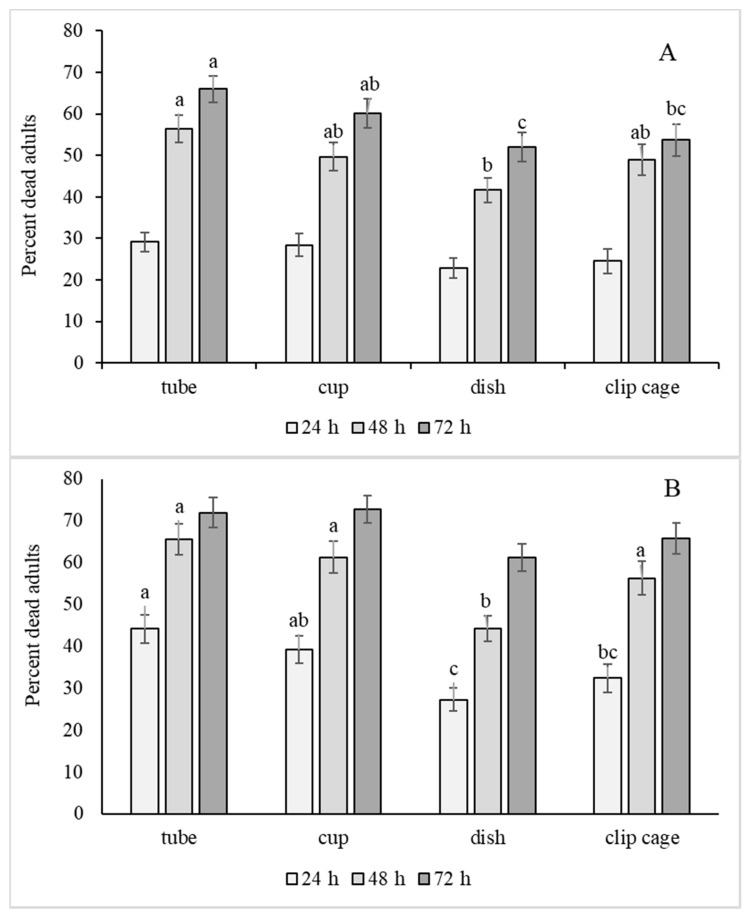
Mean % adult mortality (±se) of Florida (**A**) and Georgia (**B**) populations for tube, cup, petri dish, and clip cage methods at 24, 48 and 72 h. Means within hour reading labeled with the same lower case letter or no letter were not significantly different (LSD, *p* < 0.05).

The significant hour x bioassay method interaction for percent mortality was difficult to discern, but appears to be related to differences in bioassay response between hour readings (Figure 6). For example, the dish method had a clear stair-step change in response over time; this was similar to that seen in the overall mean in Figure 5. However, the tube and clip cage methods had the greatest change in response between 24 and 48 h and much less change between 48 and 72 h, suggesting that for these methods, waiting until 72 h does not provide as much additional response information as for the dish method. The average percent mortalities over both populations for the checks in tube, cup, dish and clip cage methods at 72 h were 26.8 ± 3.6, 25.2 ± 5.9, 23.8 ± 6.1, 29.2 ± 6.8, respectively, which were all well above the acceptable amount of check mortality for a toxicological bioassay.

Unrelated to the reading time, there were significant interactions between the bioassay method and insecticide treatment for both populations in terms of percent dead (Table 1). In the Georgia population, there was also a significant application route x insecticide interaction for percent dead (Table 1). These interactions were important in that they related to how a particular method or application route might introduce bias into the response data. The range of Tukey’s mean separation categories as indicated by lower case letters for each method was a–d (tube), a–e (cup), a–d or a–c (dish) and a–f or a–e (clip cage) (Table 4). A greater range suggested more sensitivity across treatments, so with this assumption, the clip cage method provided the most and the dish method the least sensitivity. As an example, the clip cage and cup methods separated out the high rates of the three top insecticides from the high rate of imidacloprid, whereas the dish and tube methods did not. The tube and the cup methods ranked treatments similarly (Table 4), whereas the dish had an odd response for the low rate of flupyradifurone (Table 4). The interaction between application route and insecticides related to check mortality and the range of response for each route, i.e., 14.6 ± 2.9% and 26.7 + 4.1% dead in the check and 81.8 + 3.8% and 92.7 + 2.2% dead in the best insecticide treatment, cyantraniliprole, for the root drench and leaf dip routes, respectively. Root drench treatments tended to produce drier test leaves which in turn reduced mortality in the checks. Finally, there were no significant interactions in either population for egg numbers (Table 2) or nymphs (Table 3).

## 4. Discussion

Monitoring insecticide resistance in *B. tabaci* has been critical to the management of this worldwide insect pest since the early 1980′s [3,29]. Early concerns about whitefly toxicological bioassays were centered around contact toxicity as in glass vial bioassays [30] versus ingestion toxicity of systemic insecticides in leaf dip or membrane feeding studies [31]. For imidacloprid, a simple systemic treatment of leaves using a water pic was developed early on [5] which became a standard for testing systemic insecticides [13,14,15,16]. The bioassay methods used for assessing adult mortality typically used clip cages on plants or excised leaves that were either directly drenched [14] or had insecticide mixtures applied systemically through the leaf petiole [16,17]. Bioassays using treated leaves in a petri dish [17,24] or a cup [23] container have also been used. The amount of systemic insecticide absorbed by leaf petiole treatments can be high, skewing response data up compared to the mortality measured in a field application [32], but it was not clear in the literature whether systemic (root or petiole) or leaf dip would also change insecticide response values. Our results suggest that either route, through the root/petiole or directly through the leaf itself provides similar adult mortality response.

In this study, we obtained similar results between the Florida and Georgia whitefly populations which give us some confidence that the interactions observed were predictive for what might be expected for these bioassays. The similarity in response between leaf and root drenches for these insecticides was reassuring for comparisons between these types of bioassays. There are relatively few whitefly toxicological bioassay studies where multiple methods were compared in the same project, mainly to assess either adult or immature whitefly response [14,33]. Sain et al. [34] found that there was an advantage of using an intact leaf compared to multiple methods using leaf disk or detached leaf bioassays. Our results were similar in that the one detached leaf bioassay used, the dish method, provided the poorest dose response data for whiteflies. The reassuring result from our comparison is that the various methods of assessing whitefly response through bioassays is surprisingly similar, just reflecting a bit better resolution with some methods, such as the clip cage method. This means that the various bioassays employed in the literature for whitefly insecticide response are quite comparable.

## 5. Conclusions

Whitefly toxicological bioassays are critical in the development and maintenance of insecticide resistance management programs for the whitefly, *Bemisia tabaci*. We found that there is a fair amount of consistency between the various bioassay methods currently used for whiteflies, just small differences in the sensitivity to insecticide rate response. Clip cage bioassays with an intact leaf provided greater sensitivity to insecticide treatment than the petri dish method with the excised leaf. In these studies, high rates of cyantraniliprole, dinotefuran and flupyradifurone insecticides resulted in the highest incidence of adult whitefly mortality.

## Figures and Tables

**Table 1:** Effects of treatments and their interactions on adult mortality (percent dead corrected by subtracting dead adults at the time of loading the arenas) for Florida and Georgia whitefly populations, Coastal Plain Exp. Station, Tifton, GA, USA, 2019.

Treatment	Florida Population			Georgia Population		
	DF ^1^	F	P > F	DF	F	P > F
Application Route	1, 26.5	0.29	0.598	1, 23.7	0.27	0.606
Bioassay	3, 26.6	3.18	0.040	3, 23.9	8.29	<0.001
Insecticide	11, 296	41.5	<0.001	11, 354	39.1	<0.001
Hour	2, 915	222	<0.001	2, 798	338	<0.001
Application × Bioassay	3, 26.5	0.82	0.492	3, 23.2	1.95	0.149
Application × Insecticide	11, 315	1.88	0.041	11, 404	5.97	<0.001
Application × Bioassay × Insecticide	33, 315	1.15	0.269	33, 399	1.12	0.307
Bioassay × Insecticide	33, 313	1.20	0.213	33, 394	2.69	<0.001
Hour × Application	2, 911	0.03	0.969	2, 760	1.08	0.339
Hour × Bioassay	6, 914	2.21	0.040	6, 769	3.96	<0.001
Hour × Insecticide	22, 913	2.75	<0.001	22, 766	2.91	<0.001

^1^ Proc Glimmix degrees of freedom (numerator, denominator), results fitted to a normal distribution of data.

**Table 2:** Effects of treatments and their interactions on egg numbers for a Florida and Georgia whitefly population, Coastal Plain Exp. Station, Tifton, GA, USA, 2019.

Treatment	Florida Population			Georgia Population		
	DF ^1^	F	P > F	DF	F	P > F
Application Route	1, 2	0.16	0.729	1, 3	3.74	0.149
Bioassay	3, 6	3.53	0.089	3, 9	2.39	0.136
Insecticide	11, 220	6.34	<0.001	11, 264	3.12	<0.001
Application * Bioassay	3, 6	0.25	0.859	3, 9	0.38	0.773
Application * Insecticide	11, 220	0.97	0.474	11, 264	1.74	0.065
Application * Bioassay * Insecticide	33, 220	0.95	0.554	33, 264	0.58	0.968
Bioassay * Insecticide	33, 220	1.44	0.065	33, 264	0.77	0.817

^1^ Proc GLM degrees of freedom (numerator, denominator), results fitted to a normal distribution of data.

**Table 3:** Effects of treatments and their interactions on nymph numbers for a Florida and Georgia whitefly population, Coastal Plain Exp. Station, Tifton, GA, USA, 2019.

Treatment	Florida Population			Georgia Population		
	DF ^1^	F	P > F	DF	F	P > F
Application Route	1, 2	1.30	0.372	1, 3	4.12	0.135
Bioassay	3, 6	0.820	0.529	3, 9	0.78	0.533
Insecticide	11, 220	1.16	0.317	11, 264	0.91	0.527
Application * Bioassay	3, 6	1.46	0.316	3, 9	0.78	0.533
Application * Insecticide	11, 220	0.85	0.595	11, 264	0.79	0.646
Application * Bioassay * Insecticide	33, 220	0.72	0.866	33, 264	0.73	0.860
Bioassay * Insecticide	33, 220	0.87	0.673	33, 264	0.97	0.526

^1^ Proc GLM degrees of freedom (numerator, denominator), results fitted to a normal distribution of data.

**Table 4:** Percent adult mortality of the Florida and Georgia whitefly populations across insecticide treatments by tube, cup, petri dish, and clip cage bioassay methods, respectively, Tifton, GA, USA, 2019.

Method	Insecticide Treatment	Florida Population	Georgia Population
Tube	check	25.3 ± 3.15 d ^1^	13.8 ± 2.84 d
	check	13.5 ± 1.91 d	21.6 ± 2.89 d
	pyriproxyfen—low	20.7 ± 4.22 d	28.4 ± 5.40 d
	pyriproxyfen—high	21.7 ± 3.27 d	23.2 ± 3.5 d
	imidacloprid—low	32.9 ± 4.66 c,d	53.6 ± 6.62 c
	imidacloprid—high	55.4 ± 6.42 b,c	65.5 ± 6.02 b,c
	flupyradifurone—low	64.3 ± 5.73 a,b	78.0 ± 5.76 a,b,c
	dinotefuran—low	73.7 ± 5.02 a,b	85.0 ± 4.51 a,b,c
	cyantraniliprole—low	69.0 ± 5.31 a,b	79.3 ± 5.26 a,b,c
	flupyradifurone—high	82.1 ± 3.94 a	87.7 ± 4.31 a,b
	dinotefuran—high	77.2 ± 4.73 a,b	95.8 ± 1.56 a
	cyantraniliprole—high	70.7 ± 6.29 a,b	94.4 ± 2.12 a
Cup	check	9.26 ± 3.00 e	19.6 ± 3.88 e
	check	14.5 ± 3.29 d,e	23.3 ± 3.18 e
	pyriproxyfen—low	21.7 ± 5.21 c,d,e	17.3 ± 4.29 e
	pyriproxyfen—high	18.1 ± 3.57 c,d,e	45.6 ± 7.66 c,d,e
	imidacloprid—low	26.9 ± 4.98 c,d,e	44.5 ± 5.93 d,e
	imidacloprid—high	42.4 ± 5.53 b,c,d	56.8 ± 6.40 b,c,d
	flupyradifurone—low	50.5 ± 5.72 b,c	73.4 ± 6.10 a,b,c
	dinotefuran—low	67.1 ± 5.75 a,b	77.4 ± 4.91 a,b
	cyantraniliprole—low	65.4 ± 5.33 a,b	70.5 ± 7.01 a,b,c,d
	flupyradifurone—high	77.7 ± 5.54 a	85.9 ± 4.42 a
	dinotefuran—high	81.1 ± 3.99 a	89.3 ± 3.23 a
	cyantraniliprole—high	77.1 ± 4.44 a	90.5 ± 3.60 a
Petri dish	check	8.97 ± 2.52 d	25.8 ± 5.74 c
	check	13.2 ± 3.61 d	30.0 ± 7.03 b,c
	pyriproxyfen—low	25.6 ± 5.16 c,d	24.5 ± 4.75 c
	pyriproxyfen—high	27.2 ± 5.84 c,d	36.3 ± 5.94 b,c
	imidacloprid—low	14.6 ± 3.01 d	32.7 ± 5.47 b,c
	imidacloprid—high	31.6 ± 5.09 b,c,d	38.5 ± 5.88 a,b,c
	flupyradifurone—low	55.7 ± 6.22 a,b,c	57.3 ± 7.52 a,b
	dinotefuran—low	48.4 ± 5.54 a,b,c	48.4 ± 6.69 a,b,c
	cyantraniliprole—low	54.6 ± 5.73 a,b,c	49.1 ± 5.87 a,b,c
	flupyradifurone—high	62.4 ± 5.60 a,b	58.6 ± 7.09 a,b
	dinotefuran—high	61.2 ± 5.24 a	58.2 ± 4.76 a,b
	cyantraniliprole—high	65.8 ± 7.04 a	69.7 ± 6.31 a
Clip cage	check	15.4 ± 4.51 d	23.6 ± 7.01 e
	check	19.8 ± 5.52 c,d	23.8 ± 5.00 e
	pyriproxyfen—low	21.2 ± 5.49 c,d	23.1 ± 3.63 e
	pyriproxyfen—high	26.0 ± 5.19 b,c,d	35.7 ± 8.74 c,d,e
	imidacloprid—low	33.3 ± 7.89 b,c,d	31.4 ± 5.14 d,e
	imidacloprid—high	36.5 ± 6.22 b,c,d	46.0 ± 7.53 c,d,e
	flupyradifurone—low	45.7 ± 7.21 a,b,c d	51.7 ± 6.65 c,d,e
	dinotefuran—low	50.9 ± 7.23 a,b,c	64.1 ± 6.82 b,c
	cyantraniliprole—low	53.8 ± 6.34 a,b	67.1 ± 8.10 b,c,d
	flupyradifurone—high	71.9 ± 7.96 a	79.0 ± 5.79 a,b
	dinotefuran—high	66.1 ± 7.88,a	85.2 ± 4.80 a,b
	cyantraniliprole—high	72.6 ± 6.71,a	94.4 ± 3.10 a

^1^ Means within method followed by the same lower case letter are not significantly different, Tukey’s *p* < 0.05.
